# Circulating elastin crosslinking desmosines are associated with arterial wall degradation in older adults with atherosclerosis

**DOI:** 10.14814/phy2.70854

**Published:** 2026-04-14

**Authors:** Hana Inoue, Lisa Takahashi, Hirofumi Tomiyama, Arisa Araki, Toshitaka Nagao, Taishiro Chikamori, Toyonobu Usuki, Utako Yokoyama

**Affiliations:** ^1^ Department of Physiology Tokyo Medical University Tokyo Japan; ^2^ Department of Cardiology Tokyo Medical University Tokyo Japan; ^3^ Department of Materials and Life Sciences, Faculty of Science and Technology Sophia University Tokyo Japan; ^4^ Department of Pathology Tokyo Medical University Tokyo Japan

**Keywords:** atherosclerosis, desmosines, elastin crosslinker, ischemic heart disease

## Abstract

Elastin, an extracellular matrix component that contributes to vascular integrity, is progressively degraded during vascular injury including atherosclerosis. We assessed circulating desmosine (DES) and isodesmosine (IDES), elastin‐specific crosslinking amino acids, as indicators of arterial elastin degradation by isotope‐dilution liquid chromatography–tandem mass spectrometry. Thirty‐eight patients with atherosclerotic ischemic heart disease (IHD) and 30 age‐ and sex‐matched healthy controls were enrolled. Plasma concentrations of both DES and IDES were significantly higher in IHD patients than in controls. The area under the receiver operating characteristic curve for total desmosines (DES + IDES) was 0.763. Multivariable analysis revealed that traditional risk factors for atherosclerosis were not significantly associated with plasma concentrations of desmosines. These findings suggest that circulating desmosines reflect arterial wall degeneration in atherosclerosis and are independent of traditional risk factors.

## Graphical abstract

Circulating desmosines may reflect arterial wall degeneration associated with atherosclerotic disease.

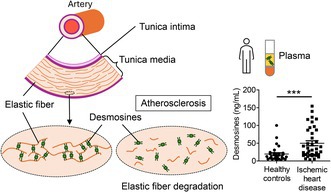

## 1 INTRODUCTION

Ischemic heart disease (IHD) remains the leading cause of death worldwide and is attributable to coronary artery stenosis, commonly associated with progression of atherosclerosis. Atherosclerosis often progresses asymptomatically, so the development of biomarkers to estimate its progression is a significant clinical challenge in assessing the risk of IHD. Because atherosclerosis is a chronic inflammatory disease, inflammation markers, including high‐sensitivity C‐reactive protein, interleukin‐6, and monocyte chemoattractant protein‐1, have been extensively investigated as biomarkers of atherosclerosis; however, these markers also reflect inflammation occurring in parts of the body other than in atherosclerotic lesions (Ajoolabady et al., 2024; Blake & Ridker, 2001; Koenig, 2013; Sproston & Ashworth, 2018).

As chronic inflammation leads to arterial wall degradation (Virmani et al., 2005; Wells et al., 2015), assessing the extent of arterial degradation may be helpful to identify patients at high risk of IHD. In this context, matrix metalloproteinases (MMP), especially MMP9, an extracellular matrix (ECM)‐degrading enzyme that is elevated in patients at high risk of plaque rupture (Galis et al., 1994; Guo et al., 2018; Johnson, 2017; Langley et al., 2017; Olejarz et al., 2020), are potential biomarkers based on arterial wall degradation in atherosclerosis. However, it has been reported that MMP9 is also markedly increased in the circulation of cancer patients, where ECM remodeling is highly active (Huang, 2018; Liang & Chang, 2018).

Elastin, a major ECM component in arterial tunica media, plays an essential role in maintaining the structural integrity, elasticity, and mechanical strength of the arterial wall (Cocciolone et al., 2018; Zhang et al., 2025). In the arteries, smooth muscle cells produce tropoelastin, which undergoes coacervation and crosslinking by the enzyme lysyl oxidase to form mature elastic fibers (Yanagisawa & Yokoyama, 2021). Desmosine (DES) and isodesmosine (IDES) are crosslinking amino acids unique to elastin (Thomas et al., 1963), thus their presence in extracellular fluids reflects elastic fiber degradation. Elevating circulating levels of DES and IDES have been reported as a biomarker in several diseases, such as chronic obstructive pulmonary disease (Ma et al., 2007; Usuki et al., 2012), abdominal aortic aneurysm (AAA) (Mordi et al., 2019), bronchiectasis (Huang et al., 2020), acute cerebral stroke (Mikagi et al., 2022), and moyamoya disease (Tashiro et al., 2024). Of these, AAA, acute cerebral stroke, and moyamoya disease are vascular diseases, so the elevation of desmosines is thought to result from arterial wall degeneration. Elevated levels of circulating desmosines have also been suggested in atherosclerosis, supported by the observation that elastic fibers are degraded within atherosclerotic lesions (Akima et al., 2009; Bizbiz et al., 1997; Nakagawa et al., 2021; Nakagawa & Nakashima, 2024; Nakashima et al., 2007; Petersen et al., 2002). Furthermore, elastin fragmentation and degradation have been reported to promote plaque development (Gayral et al., 2014; Krettek et al., 2003; Van Der Donckt et al., 2015); thus the assessment of the extent of elastin degradation is thought to be important for estimating IHD risk. However, it remains unclear whether concentrations of DES and IDES are indeed elevated in patients with IHD compared with healthy controls.

In this study, we quantified plasma DES and IDES concentrations in patients with IHD and healthy controls using isotope‐dilution liquid chromatography–tandem mass spectrometry (LC–MS/MS), which can be assigned as a powerful tool to perform quantitative analysis with high sensitivity and selectivity (Ciccimaro & Blair, 2010). We then evaluated the relationship between total desmosine concentrations and clinical characteristics, including traditional risk factors for atherosclerosis.

## 2 MATERIALS AND METHODS

### 2.1 Study participants

From April 2020 to July 2021, 38 patients who underwent catheter intervention or coronary artery bypass surgery at Tokyo Medical University Hospital were enrolled as the IHD group in this study. Plasma from participants in the IHD group was taken within 1 week before the patients received catheter intervention or coronary artery bypass surgery. Plasma samples from 30 control individuals were collected from age‐ and sex‐matched healthy volunteers at Maebashi Hirosegawa Clinic from June 2013 to January 2015. The control participants had no history of hypertension, dyslipidemia, diabetes mellitus, IHD, aortic aneurysm, cerebrovascular disease, cancer, or renal insufficiency.

Smoking status, hypertension, dyslipidemia, and the use of statins, beta‐blockers, angiotensin‐converting enzyme (ACE) inhibitors, angiotensin receptor blockers (ARBs), calcium channel blockers (CCBs), and diuretics are documented in Table 1. Body mass index (BMI) and systolic and diastolic blood pressures were measured at the time of plasma sample collection.

**TABLE 1:** Baseline characteristics of Control and IHD groups.

Variables	Control group (*n* = 30)	IHD group (*n* = 38)	*p* value
Age, years	70.7 ± 4.0	71.7 ± 5.3	0.466
Male sex, *n* (%)	25 (83.3)	31 (81.6)	1.000
Body mass index	23.1 ± 1.4	23.7 ± 2.5	0.201
Systolic BP, mmHg	130 ± 92	132 ± 18	0.447
Diastolic BP, mmHg	80 ± 7	75 ± 14	0.098
HDL cholesterol, mg/dL	57 ± 17	52 ± 14	0.128
LDL cholesterol, mg/dL	119 ± 21	88 ± 27	<0.001*
Triglyceride, mg/dL	97 ± 43	134 ± 60	0.006*
HbA1c, %	5.6 ± 0.4	6.7 ± 1.0	<0.001*
Creatinine, mg/dL	0.8 ± 0.1	0.8 ± 0.3	0.160
eGFR, mL/min/1.73 m^2^	75 ± 19	72 ± 18	0.527
Hypertension, *n* (%)	0 (0.0)	34 (89.5)	<0.001*
Dyslipidemia, *n* (%)	0 (0.0)	32 (84.2)	<0.001*
Current + past smoking	17 (56.7)	27 (71.1)	0.307
Diabetes mellitus, *n* (%)	0 (0.0)	21 (55.3)	<0.001*
Statins, *n* (%)	0 (0.0)	38 (100.0)	<0.001*
ACE inhibitors or ARBs, *n* (%)	0 (0.0)	24 (63.2)	<0.001*
β‐blockers, *n* (%)	0 (0.0)	27 (71.1)	<0.001*
Calcium channel blockers, *n* (%)	0 (0.0)	28 (73.7)	<0.001*
Diuretics, *n* (%)	0 (0.0)	4 (10.5)	0.124

*Note*: Continuous variables are shown as mean ± SD, and categorical variables are expressed as number (%).

Abbreviations: ACE, angiotensin‐converting enzyme; ARB, angiotensin II receptor blocker; BP, blood pressure; eGFR, estimated glomerular filtration rate; Hb, hemoglobin; HDL, high‐density lipoprotein; IHD, ischemic heart disease; LDL, low‐density lipoprotein; *n*, number of subjects; SD, standard deviation.

* *p* < 0.05.

The proximal segments of the left coronary artery were collected from five individual autopsy donors in 2020 at Tokyo Medical University Hospital. Tissues were fixed in 4% paraformaldehyde for histochemical staining. Donor characteristics were described previously (Takahashi et al., 2021).

### 2.2 Histochemical staining

Paraffin‐embedded blocks containing coronary artery tissues were sectioned at 4 μm, and Elastica van Gieson (EVG) staining was performed on one section from each autopsy donor to evaluate the integrity of elastic fibers. The entire tissue area in each section was analyzed.

### 2.3 Measurement of plasma DES/IDES using isotope‐dilution LC–MS/MS

**FIGURE 1:**
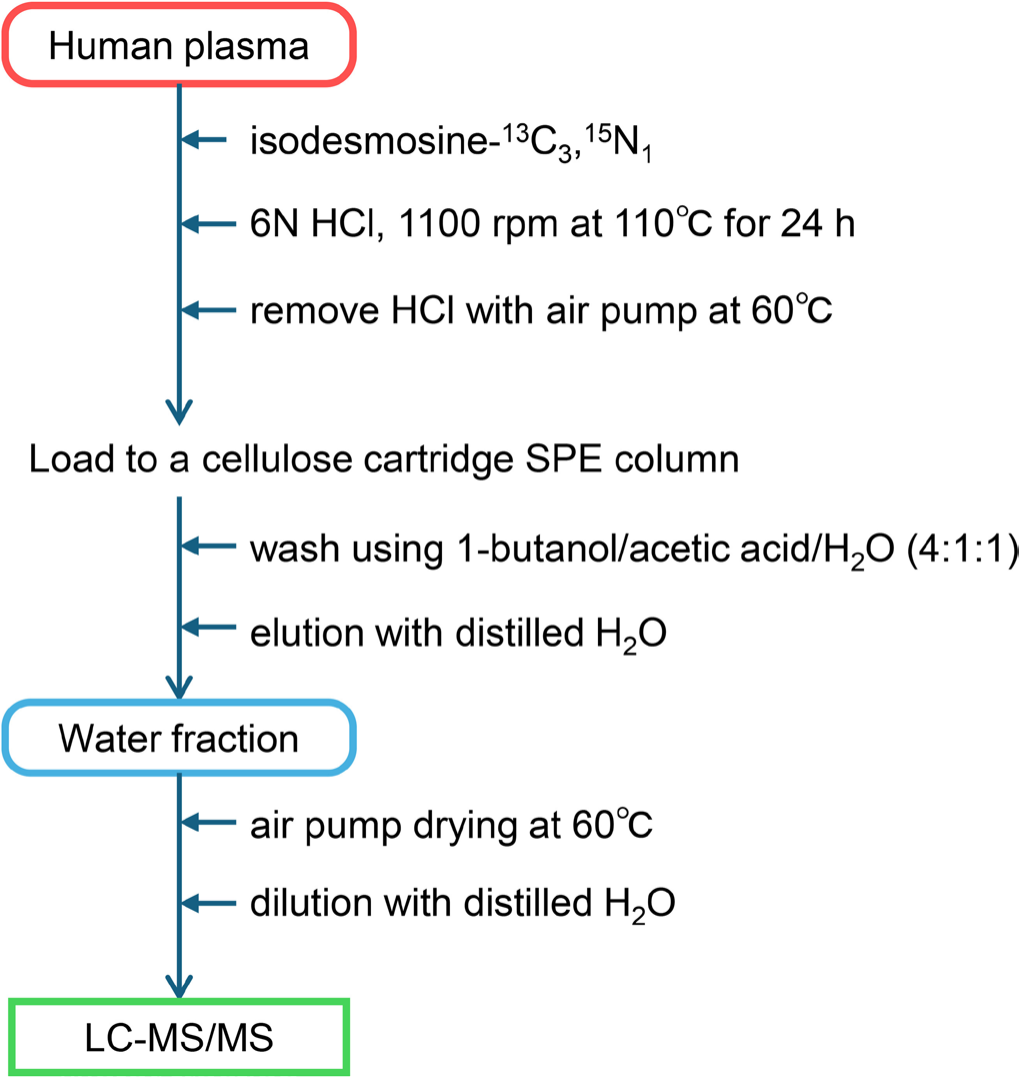
Workflow of sample preparation for analysis of desmosines by isotope‐dilution liquid chromatography–tandem mass spectrometry (LC–MS/MS).

Plasma concentrations of desmosines were determined by isotope‐dilution LC–MS/MS analysis using isodesmosine‐^13^C_3_,^15^N_1_ (IDES‐^13^C_3_,^15^N_1_) as the internal standard (ISTD). A Shimadzu LCMS8030plus quadrupole mass spectrometer (Shimadzu, Kyoto, Japan) equipped with a Discovery HS F5 column (3 μm; 2.1 mm × 15 cm) (Sigma‐Aldrich, St. Louis, MO, USA) was used for LC–MS/MS analysis. The standard compounds DES, IDES, and ISTD were originally synthesized as previously reported (Hirose et al., 2021; Tanigawa et al., 2015). Before use, the standard compounds of DES, IDES, and ISTD were purified by high‐performance liquid chromatography. Human plasma samples were pretreated as illustrated in Figure 1. Quantification was performed by selected reaction monitoring of the transitions of both DES and IDES (*m*/*z* 263.65 to *m*/*z* 232.10 + *m*/*z*397.25) and ISTD (*m*/*z* 265.65 to *m*/*z* 401.25), with collision energy set at 12.0 and 14.0 V, collision gas pressure at 1.7 mTorr, nebulizer gas flow at 2.0 and drying gas flow at 15.0 L/min, and interface voltage at 4.50 kV. The dwell time was set at 400.0 ms and the resolution of both quadrupoles (Q1 and Q3) was 0.6. The detailed conditions for LC–MS/MS analysis are shown in Tables S1, S2.

### 2.4 Statistical analysis

All statistical analyses except for receiver operating characteristic (ROC) analysis were performed using IBM SPSS Statistics (version 29.0.1.0, IBM Corp., Armonk, NY, USA). For comparison between two groups, Student's *t*‐test or unpaired Welch's *t*‐test was performed for continuous variables following an *F*‐test for equality of variances. For categorical variables, Fisher's exact test was used. Univariate analysis was conducted using linear regression. Multivariable analysis was conducted using a multiple linear regression model. To evaluate diagnostic performance, ROC analysis was performed in R (version 4.3.1) using the pROC package. The ROC curve was plotted and the area under the curve (AUC) estimated with its 95% confidence interval (CI). The optimal cutoff values were determined based on the Youden index.

## 3 RESULTS

### 3.1 Degradation of elastic fibers in the coronary arterial wall of atherosclerotic autopsy specimens

**FIGURE 2:**
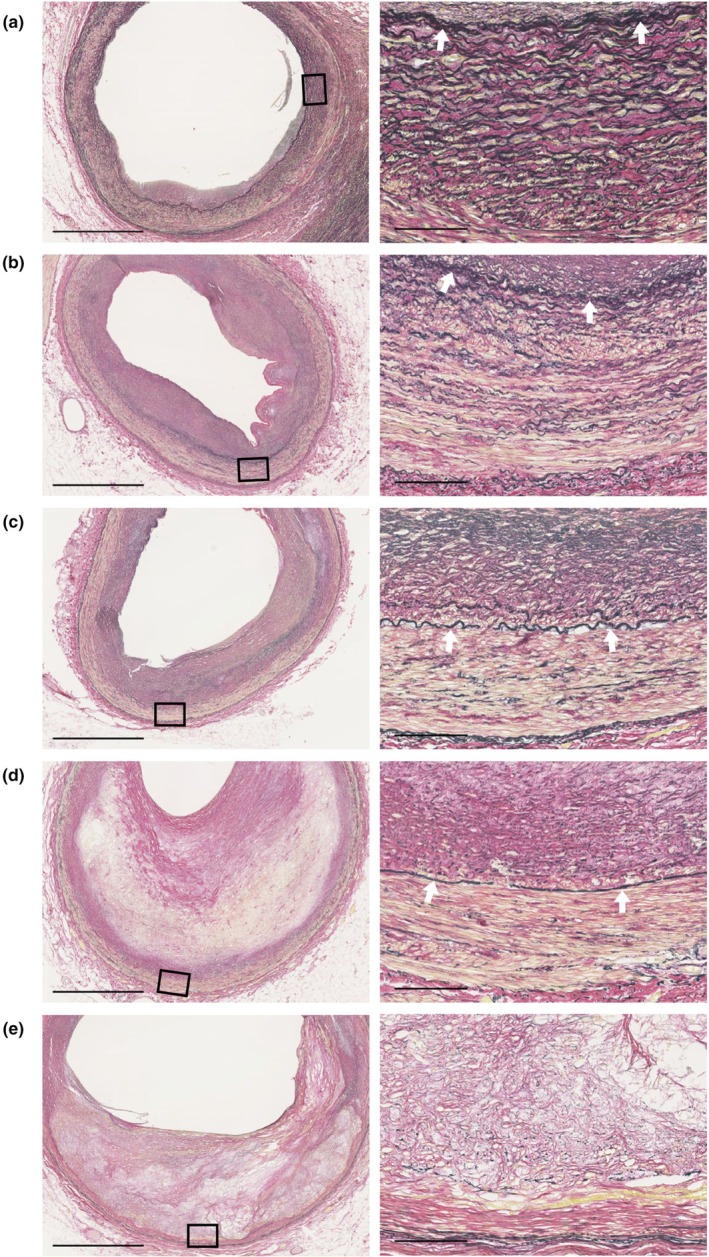
EVG‐stained images of coronary arteries obtained from 5 individual autopsy specimens. Magnified images of the boxed area in the left panels are shown in the right panels. White arrows indicate IEL. Scale bars: 1 mm (left panels) and 100 μm (right panels).

To visualize elastic fibers in atherosclerotic coronary arteries, EVG staining was performed on specimens from five individual autopsies (Figure 2). In coronary arteries with minimal atherosclerotic changes, the internal elastic lamina (IEL) was intact, and elastic fibers in the tunica media remained unaffected (Figure 2a). In early‐stage atherosclerotic lesions with intimal thickening, the IEL remained intact, whereas elastic fibers in the tunica media were reduced (Figure 2b). With disruption of IEL, characterized by discontinuity of the lamina, elastic fibers in the tunica media were further reduced (Figure 2c). In advanced atherosclerotic lesions with plaque formation and luminal stenosis, elastic fibers apparently became sparse (Figure 2d,e).

### 3.2 Characteristics of enrolled participants

Baseline characteristics of the control and IHD groups are summarized in Table 1. There were no significant differences in age, sex ratio, BMI, systolic and diastolic blood pressure, high‐density lipoprotein (HDL) cholesterol, renal function (creatinine and estimated glomerular filtration rate [eGFR]), or smoking history between the two groups. The IHD group, all of whom were taking statins, had lower levels of low‐density lipoprotein (LDL) cholesterol than the control group. Triglyceride and hemoglobin A1c (HbA1c) levels were significantly higher in the IHD group. Diabetes mellitus, hypertension, and dyslipidemia were significantly more prevalent in the IHD group than in the control group. None of the participants in the control group were taking statins, ACE inhibitors, ARBs, β‐blockers, calcium channel blockers, or diuretics.

### 3.3 Plasma concentrations of DES and IDES

**FIGURE 3:**
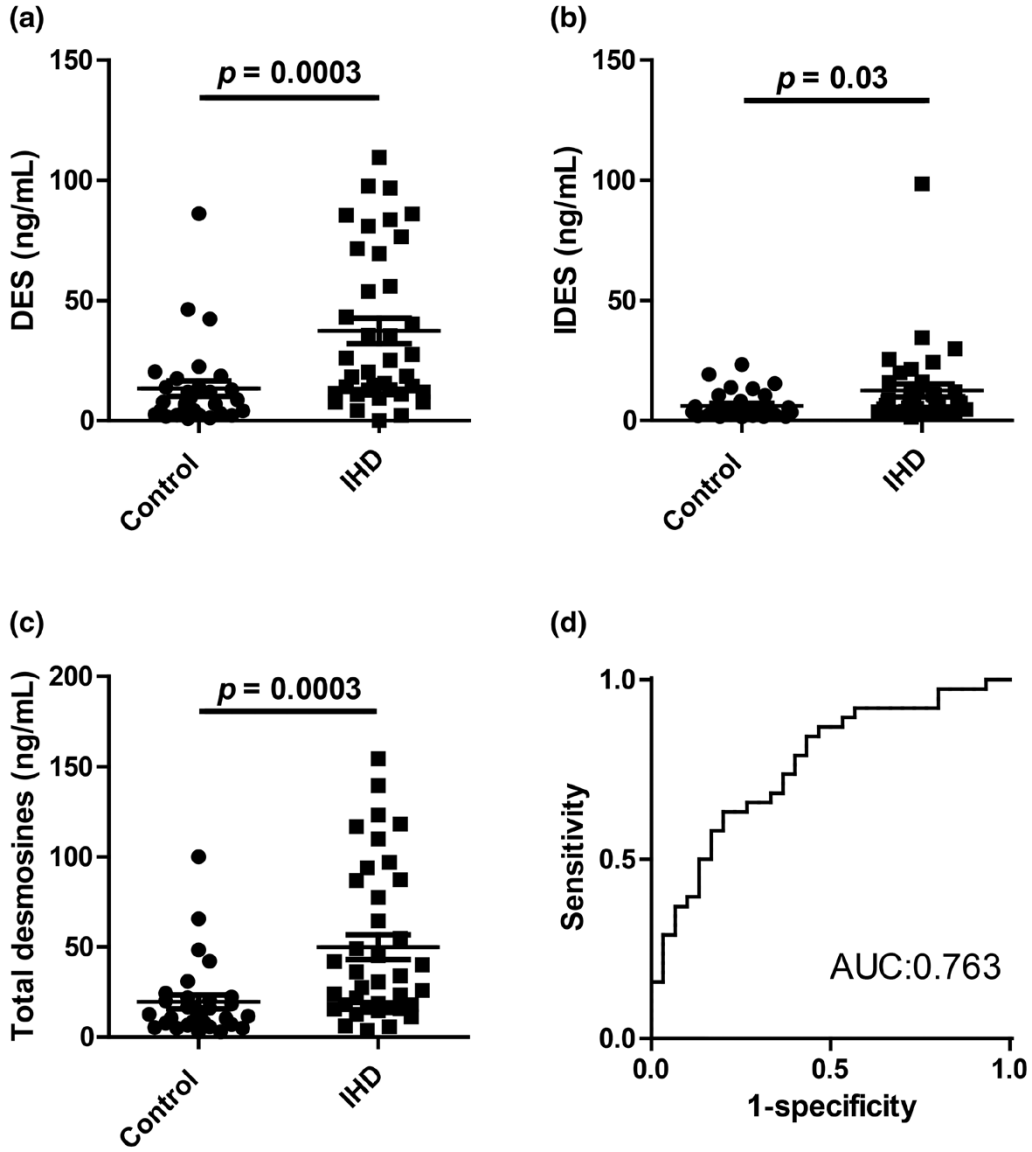
Concentrations of (a) desmosine (DES), (b) isodesmosine (IDES), and (c) total desmosines in participants with ischemic heart disease (IHD; *n* = 38) and healthy controls (*n* = 30). Unpaired Welch's t‐test. (d) Area under the receiver operating characteristic curve (AUC).

Plasma concentrations of DES and IDES were measured in both groups using isotope‐dilution LC–MS/MS analysis. DES and IDES are structural isomers of elastin cross‐links. Given that each represents only part of elastin cross‐link–derived products and that the mechanisms determining their relative formation in vivo remain unclear, DES and IDES were analyzed individually (Figure 3a,b) as well as in combination (total desmosines, Figure 3c). In our previous studies, 1 mL of plasma was typically used to prepare samples for the determination of DES and IDES concentrations. In the present study, we successfully measured DES and IDES concentrations using a smaller plasma volume (275–500 μL). Concentrations of DES were significantly higher in the IHD group than in the control group (37.3 ± 32.5 ng/mL vs. 13.3 ± 17.6 ng/mL, mean ± SD, *p* = 0.0003) (Figure 3a), and concentrations of IDES were also elevated in the IHD group (12.5 ± 16.4 ng/mL vs. 6.2 ± 5.7 ng/mL, mean ± SD, *p* = 0.03) (Figure 3b). Accordingly, the concentration of total desmosines (DES + IDES) was significantly elevated in the IHD group compared with controls (49.9 ± 42.5 ng/mL vs. 19.5 ± 20.8 ng/mL, mean ± SD, *p* = 0.0003) (Figure 3c). Thus, we used total desmosines in the subsequent analyses.

### 3.4 Utility of plasma desmosines in the detection of severe atherosclerosis

We performed ROC analysis to assess whether total desmosines could be used to detect severe atherosclerosis associated with IHD (Figure 3d). Comparing the control group with IHD group, the AUC of total desmosines was 0.763 (95% CI: 0.650–0.877), with a specificity of 80.0% at a sensitivity of 62.2%. The positive and negative predictive values were 79.3% and 63.2%, respectively, with an overall accuracy of 70.1% and a cutoff value of 22.741 ng/mL.

### 3.5 Association between total desmosine concentration and risk factors of atherosclerosis

We further investigated which traditional risk factors were associated with elevated total desmosine concentration. In the univariate analysis, total desmosine concentration was significantly associated with sex, hypertension, and dyslipidemia, which are established risk factors for atherosclerosis (Table 2). However, these associations were no longer significant in the multivariable analysis (Table 3), suggesting that plasma desmosine concentration may be independent of traditional risk factors for atherosclerosis.

**TABLE 2:** Univariate analysis between risk factors for atherosclerosis and plasma concentration of desmosines (desmosine + isodesmosine) in all participants.

Variable	*B*	95% CI	*β*	*p* value
Age	−0.735	−2.661, 1.190	−0.093	0.448
Male sex	−24.930	−48.221, −1.639	−0.254	0.036*
Body mass index	4.248	−0.015, 8.512	0.238	0.051
Hypertension	21.583	4.003, 39.162	0.289	0.017*
Dyslipidemia	28.497	11.488, 45.505	0.381	0.001*
LDL cholesterol	−0.258	−0.574, 0.057	−0.198	0.106
Smoking history	−3.335	−22.529, 15.859	−0.043	0.730
Diabetes mellitus	19.200	−0.103, 38.504	0.237	0.051

Abbreviations: B, unstandardized coefficient; β, standardized coefficient.

* *p* < 0.05.

**TABLE 3:** Multivariable analysis between risk factors for atherosclerosis and plasma concentration of desmosines (desmosine + isodesmosine) in all participants.

Variable	*B*	95% CI	*β*	*p* value
Age	−1.002	−2.902, 0.898	−0.127	0.296
Male sex	−23.520	−49.099, 2.059	−0.240	0.071
Body mass index	2.303	−1.927, 6.532	0.129	0.280
Hypertension	−7.761	−36.374, 20.852	−0.104	0.589
Dyslipidemia	25.504	−1.686, 52.695	0.341	0.065
LDL cholesterol	−0.183	−0.556, 0.189	−0.140	0.328
Smoking history	−3.686	−21.267, 19.874	−0.047	0.732
Diabetes mellitus	7.765	−14.853, 30.384	0.096	0.495

Abbreviations: B, unstandardized coefficient; β, standardized coefficient.

## 4 DISCUSSION

In the present study, we demonstrated for the first time that plasma DES and IDES concentrations were significantly elevated in IHD patients compared with healthy controls. We confirmed using autopsy specimens that elastic fiber degradation was observed in the coronary arterial wall during the progression of atherosclerosis. Our findings are consistent with previous studies suggesting that elastin degradation occurs in patients with atherosclerosis, based on histological analyses and measurements of circulating elastin‐derived peptides (Akima et al., 2009; Bizbiz et al., 1997; Nakagawa et al., 2021; Petersen et al., 2002). Elastin‐degrading enzymes such as MMPs and cathepsins, which are mainly produced by macrophages in atheroma plaques (Bizbiz et al., 1997; Galis et al., 1994; Sukhova et al., 1998), may contribute to elastin degradation, leading to elevation of circulating ECM components, including DES and IDES.

The potential of circulating or urine desmosines as a prognostic factor has been proposed in multiple disease conditions characterized by ECM degradation, including chronic obstructive pulmonary disease (Ma et al., 2007; Ongay et al., 2016; Usuki et al., 2012), cystic fibrosis (Albarbarawi et al., 2013; Laguna et al., 2009, 2012), and bronchiectasis (Hirata et al., 2024; Huang et al., 2020), vascular diseases (Ali et al., 2022; Mordi et al., 2019; Murakami et al., 2014; Tashiro et al., 2024), and cancer (Starcher et al., 2013). Among these conditions, acute myocardial infarction (AMI), a major clinical manifestation of IHD, was previously described by Ali and colleagues (Ali et al., 2022). They analyzed the clinical outcomes of AMI patients at 2 years post‐AMI and found that plasma DES concentrations were higher in patients with poor outcomes. However, they noted a limitation in that the risk predictive value of plasma DES concentrations in those AMI patients remained unknown, as their study population included patients with severe renal disease. Our study compared IHD patients without renal disease and healthy controls. Our findings suggest that the higher DES concentrations observed in AMI patients may be associated with poor outcomes even in those with renal disease. However, further studies are required to confirm this possibility.

The multivariable analysis demonstrated that plasma total desmosine concentration was independent of traditional risk factors, including hypertension (Table 3). Although hypertension is known to contribute to ECM degradation (Cai et al., 2021; Lin & Davis, 2023), no significant association between desmosine concentration and hypertension was observed in the present study. Hypertension alone may not cause ECM degradation to a level that elevates circulating desmosine concentration. In addition, as shown in Table 1, blood pressure in hypertensive patients was effectively controlled with pharmacological agents, such as ACE inhibitors, ARBs, β‐blockers, calcium channel blockers, and diuretics. This adequate control of blood pressure likely reduced hemodynamic stress on the vascular wall, which may explain in part the lack of association between desmosine concentration and a diagnosis of hypertension. Similarly, LDL cholesterol levels were well controlled in patients with dyslipidemia, which may explain the absence of an association with dyslipidemia (Table 3).

A recent study by Shek and colleagues revealed that plasma DES concentrations increase with age, regardless of the presence or absence of disease (Shek et al., 2024). Their study included participants ranging in age from their 30s to 80s. In contrast, the participants in our present study were aged between 61 and 79 years. This relatively narrow age range may have made it difficult to detect a significant association between age and DES concentration. Nevertheless, among age‐matched individuals within a 20‐year span, DES concentrations may still reflect the extent of arterial degradation due to atherosclerosis.

There are limitations to the present study. First, the sample size was small. Second, it remains unclear whether the elevation of plasma DES and IDES concentrations precedes an acute episode of IHD. Third, because all medicated subjects were exclusively included in the IHD group, medication use was completely confounded with disease status. Therefore, the present study cannot disentangle the independent effect of IHD from the effect of medication use on circulating DES and IDES concentrations. Statins have been reported to reduce MMP secretion and attenuate elastin degradation (Crisby et al., 2001; Luan et al., 2003). ACE inhibitors and ARBs may preserve arterial elastin content (Meng et al., 2015; Wojakowski et al., 2001). In contrast, CCBs have been associated with increased elastin degradation and reduced aortic elasticity in aortic aneurysm and dissection (Ma et al., 2025). Therefore, the overall effects of medication on plasma DES and IDES concentrations in the present cohort remain uncertain. Larger prospective studies are needed to determine whether plasma DES and IDES concentrations have prognostic value in atherosclerosis independent of medication effects.

In the present study, we demonstrated that circulating desmosines concentrations are elevated in patients with atherosclerosis, although the molecular basis of elastic fiber degradation in this context was not assessed. Future studies may clarify the underlying molecular mechanisms and further evaluate the relationship of circulating desmosines with arterial degeneration and disease severity in atherosclerosis.

## 5 CONCLUSION

Plasma DES and IDES could serve as promising markers of advanced atherosclerosis, reflecting arterial wall degeneration.

## AUTHOR CONTRIBUTIONS

**Hana Inoue:** Data curation; formal analysis; investigation; validation; visualization. **Lisa Takahashi:** Data curation; investigation. **Hirofumi Tomiyama:** Data curation; validation. **Arisa Araki:** Investigation. **Toshitaka Nagao:** Data curation. **Taishiro Chikamori:** Data curation. **Toyonobu Usuki:** Investigation; methodology; validation. **Utako Yokoyama:** Conceptualization; funding acquisition; supervision.

## FUNDING INFORMATION

This work was supported by Japan Agency for Medical Research and Development (AMED; JP23ek0210183 to UY), and partly supported by JSPS KAKENHI (JP23K18320 to UY).

## CONFLICT OF INTEREST STATEMENT

Hana Inoue, Lisa Takahashi, Hirofumi Tomiyama, Arisa Araki, Toshitaka Nagao, Taishiro Chikamori, and Utako Yokoyama declare no conflicts of interest. Toyonobu Usuki has received a research grant from Albion, Co., Ltd., COSE Cosmetology Foundation.

## ETHICS STATEMENT

The study protocol was approved by the Ethical Guidelines Committee of Tokyo Medical University (approval number: T2020‐0423, T2019‐0220), and the study was conducted in accordance with the principles of the Declaration of Helsinki (10th revision, Fortaleza, 2013).

## CONSENT STATEMENT

Written informed consent was obtained from all participants prior to sample collection.

## Supporting information


**Table S1.** Optimized LC conditions for DES, IDES, and IDES‐^13^C_3_,^15^N_1_.
**Table S2.** Optimized MRM mode MS/MS conditions for DES, IDES, and IDES‐^13^C_3_,^15^N_1_.

## Data Availability

The data that support the findings of this study are available upon request from the corresponding author.
