# Supplementary material for: Circulating elastin crosslinking desmosines are associated with arterial wall degradation in older adults with atherosclerosis
Source: Physiol Rep. 2026 Apr 14;14(7):e70854. doi: 10.14814/phy2.70854 (PMC13079416; doi:10.14814/phy2.70854)
Supplement: Supplementary file 1 — Table S1. Optimized LC conditions for DES, IDES, and IDES‐13C3,15N1. Table S2. Optimized MRM mode MS/MS conditions for DES, IDES, and IDES‐13C3,15N1. [file PHY2-14-e70854-s001.docx]

**Circulating elastin crosslinking desmosines are associated with arterial wall degradation in older adults with atherosclerosis**

Hana Inoue^1^, Lisa Takahashi^1,2^, Hirofumi Tomiyama^2^, Arisa Araki^3^, Toshitaka Nagao^4^, Taishiro Chikamori^2^, Toyonobu Usuki^3*^, Utako Yokoyama^1*^

^1^Department of Physiology, Tokyo Medical University, Tokyo, Japan

^2^Department of Cardiology, Tokyo Medical University, Tokyo, Japan

^3^Department of Materials and Life Sciences, Faculty of Science and Technology, Sophia University, Tokyo, Japan

^4^Department of Pathology, Tokyo Medical University, Tokyo, Japan

*Correspondence*: Utako Yokoyama, MD, PhD, FAHA, Department of Physiology, Tokyo Medical University, 6-1-1 Shinjuku, Shinjyuku-ku, Tokyo 160-8402, Japan. Tel: 81-3-3351-6141, Fax: +81-3-5379-0658, E-mail: uyokoyam@tokyo-med.ac.jp;

Toyonobu Usuki, PhD, Department of Materials and Life Sciences, Faculty of Science and Technology, Sophia University, 7-1 Kioicho, Chiyoda-ku, Tokyo 102-8554, Japan. Tel: +81-3-3238-3446, Fax: +81-3-3238-3361, E-mail: t-usuki@sophia.ac.jp

**Table S1.** Optimized LC conditions for DES, IDES, and IDES-^13^C_3_,^15^N_1_.

Column: Discovery HS F5-3 (3 μm; 2.1 mm × 15 cm) (Sigma-Aldrich, St. Louis, MO, USA)

Mobile phase: A = 0.1%FA-MeCN, B = 0.1%FA-H_2_O

0-4 min: A:B = 90:10

4-4.5 min: A:B = 90:10-10:90

4.5-7.5 min: A:B = 10:90-5:95

7.5-15 min: A:B = 5:95

15-17 min: A:B = 5:95-90:10

17-20 min: A:B = 90:10

Flow rate: 0.2 mL/min

Column temp.: 40 ºC

Retention time: 12 min

**Table S2.** Optimized MRM mode MS/MS conditions for DES, IDES, and IDES-^13^C_3_,^15^N_1_.

MRM(+)

Precursor ion (*m*/*z*): 263.65 [DES/IDES + H]^2+^

Pause time: 1.0 msec

Dwell time: 400.0 msec

Product ion: Ch1: 232.10, Q1 = -12.0 V, CE = -12.0 V, Q3 = -15.0 V

Ch2: 397.25, Q1 = -11.0 V, CE = -14.0 V, Q3 = -27.0 V

Precursor ion (*m*/*z*): 265.65 [ISTD + H]^2+^

Pause time: 1.0 msec

Dwell time: 50.0 msec

Product ion:Ch1: 401.25, Q1 = -11.0 V, CE = -14.0 V, Q3 = -27.0 V

ISTD: IDES -^13^C_3_,^15^N_1_.
